# Morphological and complete genome analysis of a novel *Clostridium perfringens* phage CpP_VarHS, isolated from wastewater

**DOI:** 10.3389/fphar.2026.1807000

**Published:** 2026-05-22

**Authors:** Hamza Saghrouchni, Semra Tasdurmazli, Ibrahim Halil Kilic, Isil Var

**Affiliations:** 1 Department of Biotechnology, Institute of Natural and Applied Sciences, Çukurova University, Adana, Türkiye; 2 Department of Molecular Biology and Genetics, Yildiz Technical University, Istanbul, Türkiye; 3 Department of Biology, Faculty of Arts and Sciences, Gaziantep University, Gaziantep, Türkiye; 4 Department of Food Engineering, Engineering Faculty, Çukurova University, Adana, Türkiye

**Keywords:** antibacterial activity, *Clostridium perfringens*, lytic bacteriophage, molecular sequencing, transmission electron microscope, phage therapy

## Abstract

**Introduction:**

Various systemic and enteric diseases have been attributed to *C. perfringens*, including healthcare-associated infections and intoxications. Humans and animals are thus exposed to serious health risks from these bacteria. Infections caused by these antibiotic-resistant bacteria are becoming an increasing concern. Bacteriophages (phages), which possess specific lytic activity, offer a viable alternative for the treatment of *Clostridium perfringens*.

**Methods:**

In this study, a novel lytic *C. perfringens* phage designed as CpP_VarHS was isolated from wastewater and targeted the host *C. perfringens* type A ATCC 13124. The phage was then characterized using Transmission Electron Microscope (TEM), molecular sequencing, and bioinformatics tools.

**Results:**

Transmission electron microscopy (TEM) analysis revealed that CpP_VarHS possesses a short, non-contractile tail (31.55 ± 2.5 nm in length) and a regular icosahedral head (44.23 ± 2.1 nm in diameter), characteristic of the podovirus morphotype. Whole-genome analysis showed that the CpP_VarHS genome is 17,814 bp long, linear double-stranded DNA with a GC content of 28.30%. Among the 21 identified open reading frames, 12 were functional genes (GenBank accession number PP973485). Whole-genome sequence comparison showed 94.37% and 94.31% nucleotide identity with *Clostridium* phage vB_CpeP_HN02 and *Clostridium* phage CPS1. No tRNA, bacterial virulence gene, lysogenic gene, or antibiotic resistance gene was identified in the genome of CpP_VarHS. Phylogenetic analysis indicated that phage CpP_VarHS represents a novel species within the genus *Gregsiragusavirus* of the family *Guelinviridae*.

**Conclusion:**

To the best of our knowledge, this is the first report of the isolation of a *C. perfringens* phage in Türkiye. In conclusion, these findings suggested that phage CpP_VarHS has promising potential for preventing and controlling *C. perfringens*.

## 1 Introduction

*Clostridium perfringens* is a Gram-positive, spore-forming anaerobic pathogen, widely distributed in the environment and responsible for various infections in humans and animals (Kiu et al., 2018). This microorganism is notably implicated in food poisoning, necrotizing enteritis, and gas gangrene, representing a significant problem in public health and animal production. Its ability to produce toxins and form spores makes it highly resistant to adverse environmental conditions and conventional antimicrobial treatments. This bacterium produces four different toxin types, which serve as the basis for classifying its strains into toxin types A to E (Kiu et al., 2018). Among them, type A is the leading cause of *Clostridium perfringens* food poisoning, accounting for more than 4 million cases worldwide annually (Van Immerseel et al., 2004). In developed countries, it is regarded as the second most significant foodborne pathogen (Kirk et al., 2015). For example, in the USA, *C. perfringens* is responsible for ∼1 million infections yearly, causing annual economic losses of ∼$400 million (Grass et al., 2013; Hoffmann et al., 2012; Scallan et al., 2011).

Faced with the rise in multidrug resistance and the limitations of current control strategies, bacteriophages (phages) are emerging as a promising alternative for preventing and treating pathogens such as *C. perfringens* (Garvey, 2020). Phages are bacterial viruses abundant in nature and capable of specifically infecting and lysing their hosts, offering a targeted, commensal microbiota-friendly approach in contrast to broad-spectrum antibiotics (Clavijo et al., 2022). Phage therapy is generally considered safe via different routes of administration, with a low incidence of side effects (Uyttebroek et al., 2022).

There are generally two principal types of bacteriophage: lytic phages and lysogenic phages. Lytic phages infect their host and cause immediate lysis, releasing new viral particles into the environment. Conversely, lysogenic phages integrate their genetic material with that of the host bacterium and can remain dormant for an extended period before entering the lytic phase. For phage therapy, only strictly lytic phages are considered suitable, as they ensure rapid and efficient elimination of target bacteria without the risk of transmitting virulence genes or antibiotic resistance (Monteiro et al., 2019). However, the isolation and characterization of new phages active against *C. perfringens* remain essential for the development of effective therapeutic strategies. Several studies focused on developing phages against *C. perfringens* strains (Seal et al., 2012).

In this study, the isolation and identification of a *C. perfringens*-specific bacteriophage CpP_VarHS from a wastewater sample were described. Then, the morphological characteristics using Transmission Electron Microscopy (TEM) and genomic analysis to assess its potential as a biocontrol agent against this pathogen were conducted.

## 2 Materials and methods

### 2.1 Sample enrichment and isolation of *Clostridium perfringens* bacteriophage

To isolate *C. perfringens* phage, 25 samples were taken from wastewater of different origins (sewer network, milk processing plant, and slaughterhouse), soil, lake water, irrigation and farm water, sheep and chicken feces, and Turkish fermented sausages (Adana, Türkiye). Briefly, 25 mL or 25 g of the sample was homogenized with 100 mL of Brain Heart Infusion (BHI) broth (Merck, Germany), inoculated with 100 μL of *C. perfringens* ATCC 13124 culture, and incubated at 37 °C overnight. After incubation, 10 mL of the culture was centrifuged at 6000 *g* at 4 °C for 10 min and filtered through a 0.45-μm pore-size sterile membrane filter (IsoLab, Germany). Then, the phage filtrate was diluted in BHI broth several times. Afterwards, the double-layer agar (DLA) method was then used to detect the presence of phages. 100 μL of each phage dilution was mixed at a 1:1 ratio with *C. perfringens* and incubated at 37 °C for 1 h. Then, 4 mL of BHI molten top agar (0.5% of agar) at 50 °C was added to the mixture and spilled onto plates of Tryptone Sulfite Cycloserine (TSC) agar (Merck, Germany). At the same time, to obtain the phage plaques, a spot test was performed by taking aliquots of 10 μL of the filtrate and dropping them on molten top agar inoculated with *C. perfringens*. Then, all plates were incubated anaerobically in the jar with the anaerocult A at 37 °C for 24 h. Dilutions of phage filtrate were mixed with *C. perfringens* in a molten BHI agar, which was poured on a TSC plate and incubated at 37 °C for 6 h under anaerobic conditions. Isolated plaques were picked up and suspended again in SM buffer to purify the phage. Purified phages were obtained after 3–5 rounds of purification at high-titer (10^10^ PFU/mL) and stored at 4 °C (Huang et al., 2022).

### 2.2 Morphological characterization of *Clostridium perfringens* phage

The negative staining method was used to examine the morphology of the phage (Melo et al., 2018). High-titer phage suspension was deposited on copper grids with a carbon-coated Formvar carbon film on a 200 square mesh nickel grid, stained with 2% uranyl acetate (pH 4.0), and examined using a Jeol JEM 1400 TEM (Tokyo, Japan) at 80 kV, and phage imaging was performed at 120,000 magnification. The Megaview III digital camera (EMSIS, Germany) was used to collect digital images.

### 2.3 *Clostridium perfringens* bacteriophage DNA extraction

The phenol/chloroform method was used for the isolation of phage genomic DNA (Melo et al., 2014). Firstly, 500 μL of the bacteriophage suspension (10^9^ PFU/mL) was subjected to an initial treatment to eliminate nucleases. Specifically, 1.25 μL of DNase I and RNase, each at a concentration of 20 mg/mL, were added, and the mixture was incubated at 37 °C for 1 h. Following this, the phage suspension was mixed with 20 μL of 0.5 M EDTA (pH 8.0, achieving a final concentration of 20 mM), 25 μL of 10% SDS (resulting in a 0.5% final concentration), and 1.25 μL of proteinase K (20 mg/mL, equivalent to 20 μg total) and left for 1 h in a water bath at 60 °C. After cooling to room temperature, an equal volume of phenol/chloroform (1:1) was added and centrifuged at 3000 *g* for 5 min at room temperature. After the centrifugation, the resulting supernatant was carefully transferred to a fresh 2-mL labeled microcentrifuge. To further purify the sample, an additional equal volume of phenol/chloroform (1:1) was added, mixed, and centrifuged as described previously. The supernatant was transferred to a fresh labeled 2-mL tube, and to precipitate the nucleic acids, the supernatant was taken and mixed with 3 M sodium acetate (1:10, pH 7.5), and 2.5 volumes of ice-cold 100% ethanol before being left in the ice for 30 min. After, the mixture was centrifuged in a benchtop microfuge for 20 min at 15,000 rpm for pellet collecting. The supernatant was carefully removed, and the pellet was washed with 70% ethanol by spinning at maximum speed for 2 min. After removing the ethanol without disturbing the pellet, the tube was left open at room temperature for 30 min to allow the ethanol to evaporate. Finally, the nucleic acid pellet was dissolved in nuclease-free dH_2_O and stored at −20 °C for further use. The concentration of DNA eluents was quantified using a Qubit 2.0 (Thermo Scientific, USA) using the Qubit dsDNA HS Assay Kit (Invitrogen, Cat. No. Q32854). The purified genomic DNA was analyzed using a 0.6% agarose gel electrophoresis system against the GeneRuler 1 kb DNA Ladder, with a range of 250 to 10,000 bp (Thermo Scientific, USA).

### 2.4 The whole genome sequencing of *Clostridium perfringens* bacteriophage

To sequence the phage’s whole genome, DNA was submitted to the Novogene company, UK (https://ocsseurope.novogene.com/oauth/login). Sequencing was performed on an Illumina platform. From the DNA samples to the final data, each step, including sample test, library preparation, and sequencing, influences the quality of the data, and data quality directly impacts the analysis results. To guarantee the reliability of the data, quality control is performed at each step of the procedure. The genomic DNA was randomly sheared into shorter fragments. The obtained fragments were then end-repaired, A-tailed, and further ligated with Illumina adapters. The resulting fragments with adapters were size-selected and PCR amplified unless otherwise specified as PCR-free before proceeding for purification. The original raw data from the Illumina platform are transformed into Sequenced Reads, known as Raw Data or RAW Reads, by base calling. Raw data are recorded in a FASTQ file, which contains sequencing reads and corresponding sequencing quality (Cock et al., 2010). The sequence quality and average scores were determined as Q SCORE (Phred score) for quality control of sequencing data. Contigs were generated using the *de novo* assembly algorithm in the trial version 24 of CLC Genomics Workbench (CLC Bio, Aarhus, Denmark), followed by manual inspection. The assembled contigs exhibited relatively uniform coverage (Melo et al., 2018).

### 2.5 Bioinformatics analysis

Once the whole sequence was obtained, and to detect and remove any vector contamination, the sequence was analysed against VecScreen (https://www.ncbi.nlm.nih.gov/tools/vecscreen/). After, the sequence was annotated using Rapid Annotation using the Subsystem Technology server (RAST) available online at (https://rast.nmpdr.org/) before being submitted to the GenBank database to obtain the genome accession number (https://submit.ncbi.nlm.nih.gov/). The sequenced genome was aligned using the Basic Local Alignment Search Tool (BLAST) available online at (https://blast.ncbi.nlm.nih.gov/) to determine the percent identity, taxonomy with the aligned phages genomes, to compare the protein sequences encoded by genes with the protein sequences in the database, and to obtain the function of the presumed protein regions (Schoch et al., 2020). To build the circular genomic map, the program Proksee (https://proksee.ca/) has been used (Turner et al., 2021). In addition, the program was used to identify the coding sequence (CDS), GC content (GC%), GC skew ((G–C)/(G + C) = values between −1 and 1), and antibiotic resistance genes (CARD). Phylogenetic relationships of the phages were inferred through whole-genome analysis using the VICTOR platform, employing sequences obtained from the NCBI nucleotide database with a minimum query coverage threshold of 70% and a sequence identity criterion of ≥90%. tRNAscan-SE v2.0 (http://trna.ucsc.edu/tRNAscan-SE/) was employed to look for the potential presence of putative transfer RNA (tRNAs) encoding genes (Chan and Lowe, 2019). VirulenceFinder was used to identify putative virulence factor genes (Tetzschner et al., 2020). The phage life cycle was predicted using PhageAI, an online tool available at https://www.phage.ai/.

## 3 Results and discussion

### 3.1 The isolation of *Clostridium perfringens* CpP_VarHS phage

*C. perfringens* has emerged as a major concern not only in animal production and food safety, but also due to its role in zoonotic transmission and antimicrobial resistance. Its association with contaminated food poses a direct threat to human health, while its impact on livestock results in significant economic losses, including reduced weight gain and increased mortality (La Mora et al., 2020). Given the limitations of conventional antibiotics and the rise of multidrug-resistant strains, the development of innovative biotechnological strategies—such as bacteriophage therapy, vaccine development, and molecular diagnostics—is urgently required to effectively control and monitor this pathogen.

**FIGURE 1:**
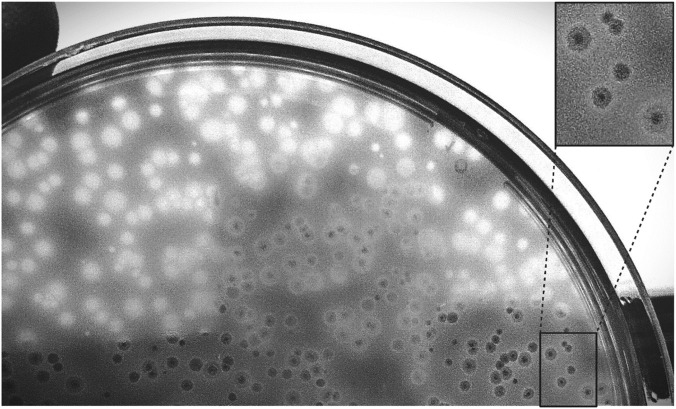
Lytic activity of the purified CpP_VarHS phage on a *Clostridium perfringens* ATCC 13124 lawn grown on TSC agar, as evidenced by the formation of clear plaques.

In this study, *C. perfringens* ATCC 13124 was used as the host strain after enrichment to isolate a lytic phage designed as CpP_VarHS from wastewater (sewer network) in Adana province in June 2023. Regarding the plaques’ morphology, after five times of purification, the phage exhibited clear, transparent, uniformly sized round plaques on the host lawn on the DLA plate, with a diameter of approximately 1 mm, with a halo of about 3 mm in diameter around the plaque (Figure 1).

The halo around the plaque of phage CpP_VarHS may be related to depolymerase. The halos can also grow in diameter with increasing incubation time (Wu et al., 2019). This protein is a putative tail fiber protein and hypothetically possesses depolymerase activity that can depolymerize the polysaccharides involved in capsular polysaccharide, exopolysaccharide, and thus biofilm formation and facilitate the access of the bacteriophage to the bacterial surfaces (host receptors). The study of Wu et al. (2019) demonstrated that a depolymerase encoded by phage showed specific enzymatic activities in the depolymerization of the capsule of *Klebsiella pneumoniae* and was able to significantly inhibit biofilm formation and degrade existing biofilms.

Regarding the isolation of phages, some phages are relatively difficult to isolate, such as *C. perfringens,* due to the anaerobic and harsh growth conditions of the host strain. Lytic phages are present in various samples, such as food, at different rates. Only a limited number of phages against obligate anaerobic bacteria have been detected, in contrast to aerobic and facultative anaerobic bacteria. Only a few lytic phages specific to clinically important species of *C. perfringens* and *Clostridium difficile* have been isolated (Hernández and Vives, 2020). Most are isolated as prophages (Ha et al., 2018).

For the isolation sources, *C. perfringens* phages from Podoviridae family (according to the former classification) have been isolated from different sources such as broiler chicken intestinal contents (Volozhantsev et al., 2011), raw sewage from a human waste treatment facility (Morales et al., 2012), livestock waste treatment plants (Ha et al., 2018), chicken meat (Noor Mohammadi et al., 2022), sewage of a donkey farm (Tang et al., 2023), chicken feces (Xuan et al., 2023), cow feces (Thanki et al., 2024), and sewage from pig farm (Wu et al., 2025).

### 3.2 Morphological characterization of *Clostridium perfringens* phage

**FIGURE 2:**
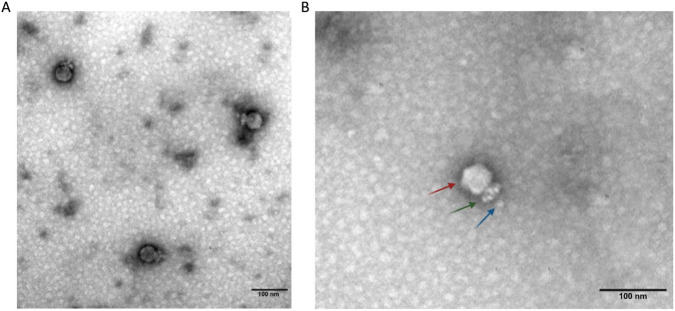
Morphological characteristics of phage CpP_VarHS. **(A)** Transmission electron micrograph of CpP_VarHS phage particles, which resemble tadpoles in shape. **(B)** The red arrow indicates the head, the green arrow indicates the tail, and the blue arrow indicates the tail knob. The sample was viewed at a magnification of 120,000×; the Scale bar represents 100 nm.

The morphological observation of the CpP_VarHS phage under TEM showed that the phage has a head (44.23 ± 2.1 nm in diameter) in the form of an icosahedron containing a short and non-contractile tail of approximately 31.55 ± 2.5 nm in length and a collar structure formed by neck appendages (Figure 2). Accordingly, we suggest that the phage belongs to the *Guelinviridae* family (Podoviridae was named before 2022), according to the International Committee on Taxonomy of Viruses (ICTV) (Smith et al., 2025; Turner et al., 2023).

This phage had a similar morphology to several known and recently reported morphologies of *C. perfringens* phages, such as vB_CpeP_15N3, vB_CP_qdyz_P5, DCp1 (Tang et al., 2023; Wu et al., 2025; Xuan et al., 2023), etc., most of which were members of the family Podoviridae (according to ICTV, abolished the Podoviridae family) that had short non-contractile tails with a collar and collar fibers.

### 3.3 The sequence analysis of the phage CpP_VarHS genome revealed that it is a new phage

**FIGURE 3:**
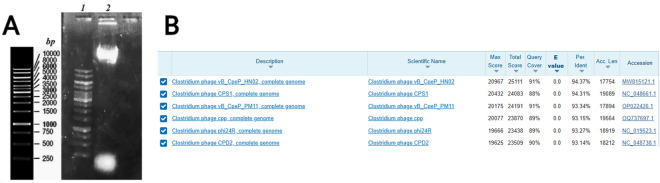
Visualization of the phage DNA integrity and sequence similarity. **(A)** Agarose gel electrophoresis profile of the genomic DNA of phage CpP_VarHS (lane 2) compared to the molecular size marker (lane 1, Thermo Scientific™ O'GeneRuler 1 kb DNA Ladder). **(B)** Genomic alignment of phage CpP_VarHS with related phages retrieved from the BLAST database, with six related phages highlighted by a red frame.

Based on Qubit measurements, the genomic DNA concentration of phage CpP_VarHS was determined to be 55.8 ng/μL. The agarose gel electrophoresis profile is shown in Figure 3A. Genome sequence analysis revealed that the phage CpP_VarHS possesses a linear double-stranded DNA of 17,814 bp (16,763,306 total reads) in length with a GC content of 28.30%. The complete genome sequence of the phage was deposited in the GenBank-NCBI database with the accession number PP973485 (submitted on 02 July 2024) and the name of *Clostridium* phage CpP_VarHS. By performing whole-genome alignment of phage CpP_VarHS with phages in the NCBI database (last accessed 08 August 2025) the results indicated that the phage CpP_VarHS had the highest similarities with *Clostridium* phage vB_CpeP_HN02 (94.37% identity, 91% query coverage) and *Clostridium* phage CPS1 (94.31% identity, 88% query coverage) (Figure 3B). They also possess short genomes (between 18,000 and 20,000 bp) and low GC contents of 28%–30% (Ha et al., 2019; Noor Mohammadi et al., 2022). The BLAST analysis results also showed the taxonomy (full lineage): *Viruses; Duplodnaviria; Heunggongvirae; Uroviricota; Caudoviricetes; Guelinviridae; Denniswatsonvirinae; Gregsiragusavirus;* unclassified *Gregsiragusavirus* with the reference NCBI:txid3234958.

Genomic analysis is an important method for identifying useful functional proteins and the safety of phage application. A total of 21 Coding Sequences (CDSs) were identified with a minimum size of 156 bp and maximum size of 2310 bp, which were transcribed on positive and negative strands (Table 1). BLASTp analysis revealed the presence of 12 proteins that showed homology with proteins with known functions and 9 hypothetical proteins, accounting for 56.52% of the functional proteins. This is due to enormous phage diversity and the limitation of the annotated genome of *C. perfringens* phages in the database. Additionally, no tRNA genes were found in the genome of CpP_VarHS, suggesting that phage functional genes strongly rely on the host for translation, and the absence of genes related to lysogeny was also determined (Ma et al., 2022). Generally, the phage genome has a modular structure, with each module containing a cluster of genes with a specific function. The following gene modules were identified: (i) DNA replication (DNA polymerase and single-strand DNA binding protein), (ii) structural proteins (portal protein, two major capsid proteins, proximal tail tube connector protein, tail protein, pre-neck appendage and lysozyme-peptidase), (iii) packaging (terminase), (iv) lysis (holin and endolysin) and (v) hypothetical proteins. The detailed information on all predicted ORFs was shown in the Supplementary file.

**TABLE 1:** Coding sequences of phage CpP_VarHS genome.

CDS	Start location	Stop location	Length (bp)	Product
CDS1	153	308	156	Hypothetical protein
CDS2	316	525	210	Hypothetical protein
CDS3	494	1213	720	Hypothetical protein
CDS4	1234	3543	2310	DNA polymerase
CDS5	3545	4567	1023	Terminase
CDS6	4673	5254	582	Single-strand DNA binding protein
CDS7	5309	5545	237	Hypothetical protein
CDS8	5691	5945	255	Hypothetical protein
CDS9	5947	7047	1101	Major capsid protein
CDS10	7062	8054	993	Major capsid protein
CDS11	8236	9612	1377	Lysozyme-peptidase
CDS12	9603	10850	1248	Tail protein
CDS13	10853	11494	642	endolysin
CDS14	11494	11667	174	Holin
CDS15	11698	12600	903	Portal protein
CDS16	12560	13264	705	Proximal tail tube connector protein
CDS17	13276	13536	361	Hypothetical protein
CDS18	13537	14934	1398	Hypothetical protein
CDS19	14957	16789	1833	Pre-neck appendage
CDS20	17157	16804	354	Hypothetical protein
CDS21	17571	17341	249	Hypothetical protein

CDS, Coding Sequence. The following gene modules were assigned: (i) DNA, replication (DNA, polymerase), (ii) structural proteins (portal protein, two major capsid proteins, proximal tail tube connector protein, tail protein, pre-neck appendage and lysozyme-peptidase), (iii) packaging (terminase), (iv) lysis (holin and endolysin) and (v) hypothetical proteins.

**FIGURE 4:**
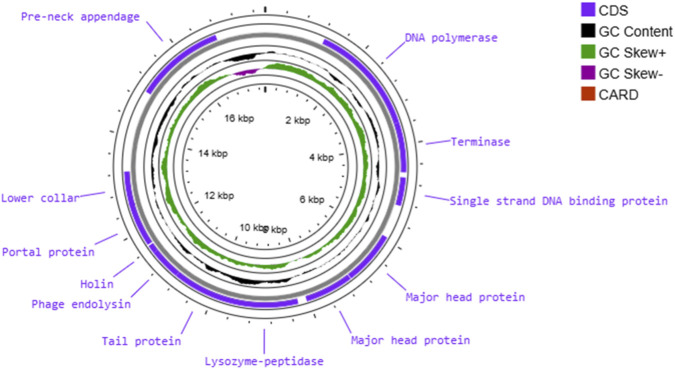
Circular genome map of phage CpP_VarHS. Circles display (from the outside): CDSs transcribed in the clockwise or the counterclockwise direction; G + C % content; (*G*–C)/(G + C) = values between −1 and 1; and CARD: The Comprehensive Antibiotic Resistance Database (CARD).

Furthermore, genomic DNA sequence analysis indicated that it is free of any predicted genes for virulence factors, antibiotic resistance genes, or toxins, indicating that CpP_VarHS is a promising and safe antibacterial agent for *C. perfringens.* The whole genome of phage CpP_VarHS is presented as shown in Figure 4. The circular map indicates the localisation of each CDS on the genome, with the G + C % content, GC skew+, and GC skew-. Two of the 21 genes were transcribed in the minus strand, and the others in the plus strand.

The CDS13 encoded endolysin, which can degrade peptidoglycan in host cell walls to inhibit or kill bacteria. Endolysin of CpP_VarHS consists of two domains, including the N-acetylmuramoyl-L-alanine amidase catalytic domain and the peptidoglycan-binding domain. N-acetylmuramoyl-L-alanine amidase (also known as peptidoglycan aminohydrolase) is an autolysin. Enzymes containing this domain can degrade the peptidoglycan by cleaving the amide bond between N-acetylmuramoyl and L-amino acids (Simmons et al., 2010). The phages that infect Gram-positive bacteria are usually host-specific, but their endolysins have a broad host spectrum (Fischetti, 2017). Further analysis is required to determine if this is the case for phage CpP_VarHS. In general, endolysins have to pass through the pores in the cell membrane created by holin, which is encoded by CDS14 (Young and Bläsi, 1995). The holin gene is most likely downstream of the lysin gene, which is the same for CpP_VarHS phage. This placement is unique among the other Clostridial bacteriophages, including the *Podoviruses* (Oakley et al., 2011). The hallmark characteristic of the existence of phage-encoded polysaccharide depolymerases is plaque-surrounding halos. However, the gene encoded for depolymerase was not identified in the CpP_VarHS genome and may be among the hypothetical or unannotated proteins. The molecular characterization of phage CpP_VarHS is important in expanding our knowledge of *C. perfringens* phages.

**FIGURE 5:**
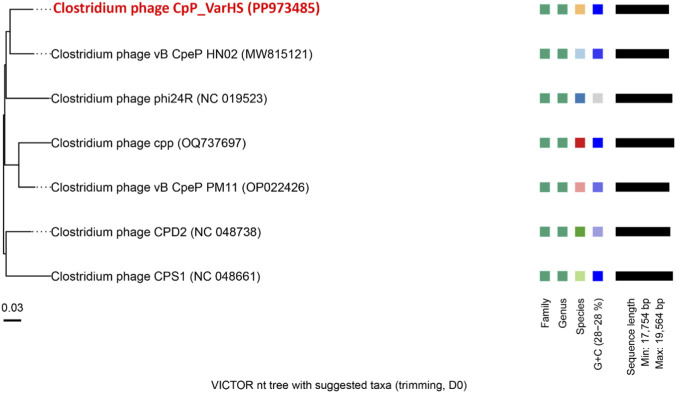
Phylogenomic reconstruction of phage CpP_VarHS and its six nearest relatives, generated through whole-genome comparison using the VICTOR platform.

The NCBI BLASTn analysis identified six phages with genomic similarity to the isolate (Figure 3B). Subsequent to this, a phylogenetic reconstruction was performed using the VICTOR platform. This reconstruction was based on whole-genome comparisons and integrated BLASTn-derived data under standardized thresholds (≥70% query coverage and ≥90% nucleotide identity). The GBDP phylogenomic tree (Figure 5), inferred using the D0 formula, displays branch lengths scaled to the intergenomic distances derived from the respective metric. Within this framework, CpP_VarHS demonstrated a high degree of similarity with *Clostridium* phage vB CpeP HN02 and several other *C. perfringens* phages, suggesting a close evolutionary relationship within this clade. Taxonomic assignment, illustrated through color coding, substantiated its classification within the same family and genus. Nevertheless, divergence at the species level indicates that CpP_VarHS represents a genetically distinct lineage. Consequently, CpP_VarHS can be classified as a novel species within the genus *Gregsiragusavirus*. Its GC content (∼28%) mirrors that of related phages, consistent with shared evolutionary adaptation to the host genome, while its genome length (∼18–19 kb) falls squarely within the established range for this group, reinforcing structural conservation. Collectively, these findings substantiate that CpP_VarHS represents a member of a cohesive cluster of *C. perfringens*-specific phages, yet exhibits sufficient divergence to warrant recognition as a new species.

Moreover, PhageAi predicted phage CpP_VarHS to be a lytic phage, which was also verified by the observation of clear phage plaques in agar plates in the experimental method. In summary, based on the whole-genome sequence alignment and phylogenetic relationship, phage CpP_VarHS represents a new virus infecting *C. perfringens* and belonging to the family *Guelinviridae*, genus *Gregsiragusavirus*. A few studies have characterized the morphology of *C. perfringens* phages, but they have revealed phages belonging to different families (Huang et al., 2022; Morales et al., 2012; Seal, 2013; Seal et al., 2011; Volozhantsev et al., 2011). Other phages belonging to the *Podovirus* morphotype are the *C. perfringens* phages (Ha et al., 2018; 2019; Noor Mohammadi et al., 2022; Tian et al., 2022). To the best of our knowledge, this is the first report on the isolation of *C. perfringens* phage in Türkiye.

The findings of this study manifested that phage CpP_VarHS is a lytic phage and lacks the genes associated with bacterial virulence and drug resistance, suggesting that phage CpP_VarHS therapy could avoid the phage-mediated transfer of virulence and drug resistance genes to the bacterial host. Additionally, the results showed that phage CpP_VarHS has an outstanding antibacterial effect *in vitro* with clear lysis plaques*.* Based on the above characteristics of the phage, we hypothesize that it may be a potential antimicrobial agent that can be used to prevent or treat the disease caused by *C. perfringens*. This study highlights that CpP_VarHS phage efficacy should be confirmed using various experiments *in situ* and *in vivo,* and provides important preliminary information for further research on the biological characterization, including its host range across *C. perfringens* strains of different origins and its stability under various environmental conditions.

## 4 Conclusion

The current study described a novel lytic *C. perfringens* phage, CpP_VarHS, isolated from wastewater and identified. Genome analysis revealed that CpP_VarHS has a linear double-stranded DNA of 17,814 bp and a G + C composition of 28.30%. Phylogenetic analysis indicated that CpP_VarHS is a novel member of the *Guelinviridae* family. The genome of the phage lacked any tRNA, virulence gene, lysogenic gene, or drug resistance gene. Thus, phage CpP_VarHS is safe for biotechnological applications. The current study provides some basic information for further research on phage CpP_VarHS and its application. Taken together, our data suggest that phage appears to be a promising therapeutic in the targeted eradication of *C. perfringens*.

## Data Availability

The datasets presented in this study can be found in online repositories. The names of the repository/repositories and accession number(s) can be found below: https://www.ncbi.nlm.nih.gov/genbank/, PP973485.
